# CENTER-IT: a novel methodology for adapting multi-level interventions using the Consolidated Framework for Implementation Research—a case example of a school-supervised asthma intervention

**DOI:** 10.1186/s43058-022-00283-5

**Published:** 2022-03-26

**Authors:** Michelle Trivedi, Shushmita Hoque, Holly Shillan, Hannah Seay, Michelle Spano, Jonathan Gaffin, Wanda Phipatanakul, Milagros C. Rosal, Arvin Garg, Lynn B. Gerald, Sarabeth Broder-Fingert, Nancy Byatt, Stephenie Lemon, Lori Pbert

**Affiliations:** 1 Division of Pulmonary Medicine, Department of Pediatrics, University of Massachusetts Chan Medical School, S5-828, 55 Lake Ave N, Worcester, MA 01655 USA; 2 Department of Population and Quantitative Health Sciences, University of Massachusetts Chan Medical School, S5-828, 55 Lake Ave N, Worcester, MA 01655 USA; 3 University of Massachusetts Chan Medical School, Worcester, MA USA; 4 Division of Pulmonary Medicine, Boston Children’s Hospital, Harvard Medical School, Boston, MA USA; 5 Division of Asthma, Allergy, and Immunology, Boston Children’s Hospital, Harvard Medical School, Boston, MA USA; 6 Department of Pediatrics, University of Massachusetts Chan Medical School, Worcester, MA USA; 7 Department of Health Promotion Sciences, University of Arizona Mel and Enid Zuckerman College of Public Health, Tucson, AZ USA; 8 Asthma and Airway Disease Research Center, University of Arizona, Tucson, AZ USA; 9 Department of Psychiatry, University of Massachusetts Chan Medical School, Worcester, MA USA; 10 Department of Obstetrics and Gynecology, University of Massachusetts Chan Medical School, Worcester, MA USA

**Keywords:** Recipient, Deliverer, Patient-centered, Systems-level, Multi-level partner engagement, Implementation, Asthma, Schools, Stakeholder engagement, Evidence-based interventions

## Abstract

**Background:**

Implementation science frameworks advise the engagement of multi-level partners (at the patient, provider, and systems level) to adapt and increase the uptake of evidence-based practices (EBPs). However, there is little guidance to ensure that systems-level adaptations reflect the voices of providers who deliver and patients/caregivers who receive EBPs.

**Methods:**

We present a novel methodology, grounded in the Consolidated Framework for Implementation Research (CFIR), which anchors the engagement of multi-level partners to the voices of individuals who deliver and receive EBPs. Using the CFIR domains: *intervention adaptation*, *individuals involved*, *inner/outer setting*, and *process*, we illustrate our 4-step methodology through a case example of Asthma Link, a school-supervised asthma management intervention. In step 1, we interviewed “*individuals involved*” in the intervention (providers/caregivers/patients of Asthma Link) to identify implementation barriers. In step 2, we selected systems-level partners in the “*inner and outer setting*” that could assist with addressing these barriers. In step 3, we presented the barriers to these systems-level partners and conducted semi-structured interviews to elicit their recommended solutions (*process*). Interviews were audio-recorded, transcribed, and open-coded. A theoretical sampling model and deductive reasoning were used to identify solutions to implementation barriers. In step 4, we utilized multi-level input to *adapt* the Asthma Link *intervention*.

**Results:**

Identified barriers included inability to obtain two inhalers for home and school use, inconsistent delivery of the inhaler to school by families, and challenges when schools did not have a nurse. Interviews conducted with school/clinic leaders, pharmacists, payors, legislators, and policymakers (*n*=22) elicited solutions to address provider and patient/caregiver-identified barriers, including (1) establishing a Medicaid-specific pharmacy policy to allow dispensation of two inhalers, (2) utilizing pharmacy-school delivery services to ensure medication reaches schools, and (3) identifying alternate (non-nurse) officials to supervise medication administration. The iterative *process* of engaging multi-level partners helped to create an adapted Asthma Link intervention, primed for effective implementation.

**Conclusions:**

This novel methodology, grounded in the CFIR, ensures that systems-level changes that require the engagement of multi-level partners reflect the voices of individuals who deliver and receive EBPs. This methodology demonstrates the dynamic interplay of CFIR domains to advance the field of implementation science.

Contributions to the literature

- While many implementation science frameworks call for multi-level partner engagement, there is little guidance on how to ensure that this process reflects the voices of the providers who deliver and patients and caregivers who receive evidence-based interventions.
- We describe a simple and novel methodology: CENTER-IT, grounded in the Consolidated Framework for Implementation Research (CFIR), which ensures that the voices of providers and patients/caregivers are reflected in systems-level adaptations to evidence-based interventions.
- This methodology demonstrates an empiric use of the CFIR and shows the dynamic interaction between the five CFIR domains to inform implementation of an evidence-based school-supervised asthma intervention.

## Background

Implementation frameworks, including the Consolidated Framework for Implementation Research (CFIR), call for the involvement of multi-level partners (at the patient, provider, and systems level) throughout the implementation research process to adapt interventions and promote effective implementation and sustainability of evidence-based practices [1–3]. However, there is little guidance on how to ensure that changes at the systems-level integrate multi-level perspectives, including those of providers who deliver interventions and patients and caregivers who receive and utilize interventions.

Multi-level partners have been increasingly involved in the process of adapting evidence-based interventions (EBPs) for effective implementation [4, 5], yet systems-level partners often make decisions without input from those individuals directly affected by interventions. The voices of providers and patients need to be heard by systems-level partners, to promote decisions and adaptations that overcome implementation barriers on the ground level [6]. Recognizing this, “patient needs and resources” has been proposed as its own domain in a pragmatic adaptation to the CFIR [7]. Incorporating the voices of both the deliverers and recipients of interventions, specifically with user-centered design, improves provider delivery, receptivity, and patient uptake of EBPs [8]. Theoretical approaches depict engagement as a process that is key to implementation without clearly defining the term “engagement.” The CFIR places “engagement” within several domains (*process*, *individuals involved*, *inner and outer setting*); however, there is little explanation on how one domain influences another [4]. Moreover, within the CFIR, little research has been done to gain an understanding of the dynamic interplay between individuals and the organization in which they work and how that interplay influences individual or organizational behavior [1].

To address these gaps, we present a new methodology: CENTER-IT (CENTERing multi-level partner voices in Implementation Theory) which is grounded in the CFIR. The five CFIR domains are characteristics of the *intervention*, characteristics of the *individuals involved*, *inner setting*, *outer setting*, and *process* [1]. The CENTER-IT approach provides guidance on how to engage multi-level partners to connect all five CFIR domains and anchors the engagement of multi-level partners to the voices of individuals who deliver and receive EBPs. The purpose of this study is to describe the CENTER-IT methodology and how it was used to adapt an evidence-based intervention to promote implementation, based on stakeholder perspectives of implementation determinants at multiple levels.

## Methods

### Case study: school-supervised asthma therapy for children

In this case study, the CENTER-IT methodology is exhibited through multi-level partner engagement in the implementation of school-supervised asthma therapy. School-supervised asthma therapy is an EBP that has improved preventive medication adherence and asthma symptoms among children who are in racial/ethnic minority groups and low-income socioeconomic conditions [9–12]. However, this strategy has not been widely adopted in practice to produce meaningful public health impact for these populations. Asthma Link is a school-supervised therapy intervention in central Massachusetts, developed to increase the uptake of this EBP, in part by leveraging the existing infrastructure of pediatric practices and school nurses, rather than research resources, to operate. The details of this intervention have been previously published [13]. In brief, pediatric providers identify children with poorly controlled asthma in their practice and send medication orders to the child’s school to initiate school-supervised therapy. Families are asked to bring a preventive inhaler into the school and school nurses supervise its administration, ensuring daily adherence [13]. Approximately sixty to one hundred children with poorly controlled asthma are enrolled in Asthma Link each school year and enrolled children experience significant decreases in emergency room visits, hospital admissions, and rescue medicine use [13]. While patients/caregivers, medical providers, and school nurses have found this intervention acceptable, they also identified barriers to successful implementation in practice [14].

This research is responding to the problem that most pediatric practices are not implementing school-supervised therapy and those that are implementing this EBP do not enroll a high number of participants. The desired outcomes would be to increase pediatric practice and patient participation in the evidence-based practice of school-supervised asthma therapy, through using Asthma Link.

The goal of this case study is to describe our CENTER-IT methodology and how it elucidates the interaction between CFIR domains when working to improve implementation of EBPs. We will describe the CENTER-IT methodology, as it was applied to the Asthma Link case study, to demonstrate the process that ensures that recipient (patient/caregiver) and deliverer (school nurse and medical provider)-identified implementation barriers are used to guide systems-level partner engagement. This engagement process is intended to inform adaptations to the intervention which overcome and address these implementation barriers.

We chose the CFIR as the framework to tailor this intervention because it is a comprehensive framework for determining barriers and facilitators to multi-level interventions, draws particular attention to the intervention setting (both inner and outer setting), guides implementation of evidence-based practices from the phases of design to evaluation, and specifically has been effectively used for adaptation of care model designs, including those for asthma [1, 7, 15, 16]. We defined our study components according to the five CFIR domains, *intervention characteristics*, characteristics of *individuals involved*, *inner setting*, *outer setting*, and *process*, and associated constructs [1]. The CFIR domain “intervention characteristics” corresponds to our asthma intervention as described above, and the construct “adaptability” within this domain corresponds to the degree to which our intervention can be tailored to meet local needs. The CFIR domain “characteristics of individuals involved” and the construct “knowledge and beliefs about the intervention” correspond to the providers (pediatric providers and school nurses) who deliver and the patients/caregivers who receive school-supervised asthma therapy and their perceived barriers and facilitators to implementation. The CFIR domain “inner setting” and construct “structural characteristics” correspond to the organizations of the clinics and the schools where this intervention is implemented. The “outer setting” domain and construct “external policies and incentives” correspond to the market, policy, and legislative context which influence the operation of the intervention. Collectively, the inner and outer setting are defined as the systems level. The CFIR domain “process” and the construct “engaging opinion leaders and external change agents” correspond to the dynamic process of engagement of multi-level partners to create an “adapted intervention” which is primed for effective implementation.

### Four-step CENTER-IT methodology for multi-level engagement (Fig. 1)

#### Step 1: interview providers who deliver and patients/caregivers who receive EBPs to identify implementation barriers (CFIR domain: individuals involved)

The results of this first step in our methodology were previously published [14]. In brief, we interviewed Asthma Link patients and their caregivers, pediatric providers, and school nurses. Barriers reported included medical providers not having time to identify potentially eligible patients during clinical encounters, inability of families to obtain a second inhaler for school, challenges with delivery of the second inhaler to the school, absences in school nurse coverage to supervise medications, and concerns about dissemination and sustainability of the program [14].

**Fig. 1:**
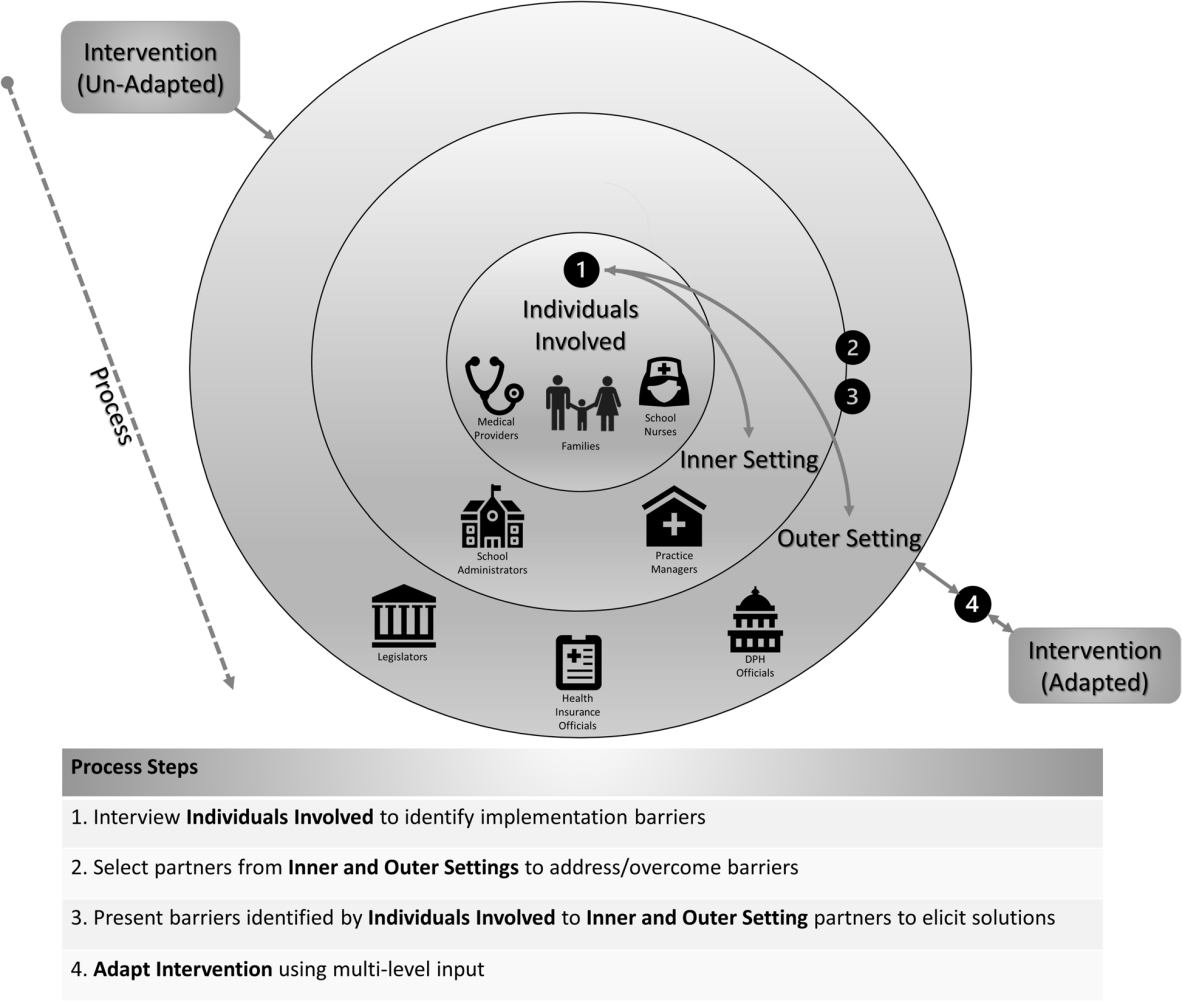
CENTER-IT: centering multi-level partner voices in implementation theory—empiric use of the CFIR

#### Step 2: select partners in the inner and outer setting of the intervention to interview, based on barriers identified by providers and patients/caregivers (CFIR domains: inner and outer setting)

An expert consensus group was created which included 2 pediatric pulmonologists and 4 behavioral scientists. Based on the provider- and patient/caregiver-identified barriers, this expert consensus group used purposive selection to determine which stakeholders in the inner and outer setting could assist with systems-level solutions to address these barriers (CFIR domain: inner and outer setting, constructs: structural characteristics and engaging opinion leaders and external change agents). The research team contacted the following systems-level groups to request interviews in the *inner setting*: five school administrators selected from the three schools enrolling the highest numbers of Asthma Link participants, four pediatric practice managers from two pediatric practices implementing Asthma Link, and four pharmacists from the most highly utilized four pharmacies. We contacted the following groups in the *outer setting*: health insurance officials including three officials in Medicaid payment services, the most highly utilized insurance by Asthma Link participants, plus one official in payment services in a private insurance company that was next highly utilized; three Massachusetts legislators with experience in child health intervention policy and child health equity; and five officials from the Massachusetts Department of Public Health with roles in early education and school-based health at the state level.

Research staff approached these partners via phone or email to request time for an interview. A maximum of two emails or phone calls were made; participation in the interview was voluntary and no compensation was provided.

#### Step 3: present implementation barriers identified by the individuals involved in the intervention, to partners in the inner and outer setting to elicit their recommended solutions (CFIR domains: process, individuals involved, inner and outer setting)

We presented the provider- and patient/caregiver-identified barriers to the partners selected from the *inner* and *outer setting* in step 2 and conducted semi-structured interviews with them to elicit their recommended systems-level solutions to these implementation issues.

We developed an interview guide for these systems-level stakeholders with fixed open-ended questions and probes to elicit input (Table 1). We developed questions within ten constructs, derived from the previously identified barriers: (1) *Asthma Link Awareness*, (2) *Completing Asthma Link Enrollment*, (3) *Current Practice*, (4) *Receiving Medications at School*, (5) *Obtaining Two Inhalers*, (6) *Delivery of Inhaler to School*, (7) *Other Asthma Link Implementation Barriers*, (8) *Stakeholder Priorities*, (9) *Dissemination of Asthma Link*, and (10) *Sustainability of Asthma Link.*

**Table 1:** Use of CFIR domains to select systems-level partners and develop interview questions for the CENTER-IT methodology: Asthma Link case study

Barriers identified by recipients and deliverers of intervention in step 1	Systems-level partners selected by expert consensus group for interviews in step 2	Example interview questions
***CFIR domain: individuals involved***	***Inner and outer Setting***	***Process***
Medical providers did not have time to identify potentially eligible patients	*Practice managers* interviewed to establish optimal procedures for identifying eligible patients	“Some medical providers have said they do not have time to identify whether a patient is eligible for Asthma Link during their clinical encounter, and therefore do not offer the program. Do you have any ideas to address this issue?”
Children and caregivers identified challenges receiving two inhalers from pharmacy	*Health Insurance Officials* and *Pharmacists* interviewed to establish protocols	“A requirement of the program is that 2 inhalers be provided: one for school and one for home. How would this best be done? Is there any special process you would recommend?” “Some families could not pick up two inhalers because their insurance would not cover the cost, despite the total number of inhalers used throughout the year being the same. What suggestions do you have to improve this process?”
School nurses identified challenges ensuring child comes to school health office daily	*School Administrators* interviewed to establish optimal procedures	“Some school nurses have said that a child may forget to come to the school health office every day. Do you have any ideas to address this issue?”
Medical providers, school nurses, children, and caregivers had concerns that other families, pediatric practices, and schools may not know about Asthma Link	*DPH Officials* and *Legislators* interviewed to establish advocacy and dissemination procedures and strategies for policy change/funding	“Some participants had concerns about how to promote the spread of this program. What ideas do you have regarding dissemination and sustainability for a program like Asthma Link at a statewide and nationwide level? What role might your organization play in this process?”

##### Data collection

Researchers trained in qualitative methods conducted semi-structured interviews over the telephone between November 2019 and June 2020. The researchers had no established relationships with the study participants. A fact sheet was reviewed with the participant and interviews were conducted in a private setting. The interview guide contained a mix of open- and closed-ended questions. For each systems-level group, we asked questions related to the specific barriers identified by Asthma Link participants to seek input on optimal ways to overcome and address these challenges (Table 1). Probes and follow-up questions were used throughout the phone interview as needed. Each interview lasted between 30 and 45 min and all were audio recorded. The Institutional Review Board at the University of Massachusetts Medical School approved this study.

#### Step 4: adapt intervention protocol using multi-level input

Finally, we incorporated this multi-level input to understand implementation determinants at multiple levels and develop adaptations to the Asthma Link intervention protocol. An expert consensus group was created which included two pediatric pulmonologists and four behavioral scientists, who examined this input from partners on the implementation determinants at multiple levels and utilized this data to inform adaptations to the intervention. This expert consensus group closely considered the risks of creating adaptations to the intervention, with regard to compromising fidelity, effectiveness, and appropriateness of the intervention with attention to the compromise between fidelity and fit of these adaptations [17].

### Qualitative analysis

A third party transcribed the recorded interviews, checked for accuracy, and stripped identifying information. Four members of the research team (SH, MS, HS, HS), trained in qualitative analysis, performed thematic content analysis using a deductive approach, guided by the 10 a priori defined constructs [14, 18]. The CFIR was an integral part of our analysis; within each a priori construct, specific CFIR constructs guided the content analysis. An iterative, constant-comparative process was used to review, segment, and open code data into emerging themes and recurrent patterns [19]. Four team members (SH, MS, HS, HS) read five randomly selected transcripts as a group line-by-line and assigned preliminary codes to each unique topic that emerged. Following review of these transcripts, the research team refined these codes and specified indications for their use in a universal codebook [18, 20]. The remaining transcripts were coded individually by the same four team members based on the universal codebook. All coding discrepancies were resolved through consensus. Qualitative data was organized and analyzed using Dedoose software, version 8.3.17 (2020) [21]. Each team member scored greater than 80% on Dedoose coding tests, resulting in Cohen’s kappa of 1.0 and supporting interrater agreement [22, 23].

After completion of open coding, the research team used deductive thematic analysis to identify major themes according to the 10 a priori defined constructs [24], based on the previous provider- and patient/caregiver- identified barriers. Within these constructs, researchers looked for alignment in themes within and across systems-level groups and analysis continued until no new themes emerged eluding to saturation [25]. To ensure that the final themes accurately reflected the perspectives of interviewees, themes were validated by member checks: one interviewee from each of the systems-level groups was contacted by phone to present thematic results and to provide an opportunity for them to suggest changes [26, 27]. All of these partners unanimously agreed on the presented themes. The themes that informed adaptations to the Asthma Link protocol are described below.

## Results

### Study population

We contacted 25 systems-level partners from the inner and outer setting and 22 (88%) agreed to participate in the key informant interviews. These included Department of Public Health officials (*n* = 4), school administrators (*n* = 4), pediatric practice managers (*n* = 3), health insurance officials (*n* = 4), pharmacists (*n* = 4), and legislators (*n* = 3).

### Data constructs and themes

Within the 10 a priori defined constructs, we identified 17 sub-constructs based on the qualitative input. Major themes that emerged were listed within each sub-construct. Key examples of constructs (barriers) and themes (recommended solutions) are described below.

### Interactions of CFIR domains within the CENTER-IT methodology

Figure 1 presents the dynamic interaction between the five CFIR domains within the CENTER-IT methodology: *individuals involved*, the *inner and outer setting*, the *process* of engagement, and how the use of this methodology led to *intervention adaptations* to improve implementation. The findings presented below demonstrate the key products of the CENTER-IT methodology. We show the results of our 4-step process in Table 2, according to each previously identified implementation barrier: (1) the providers/patients/caregivers that identified each barrier in our previous study (*CFIR domain: individuals involved*), (2) the selected systems-level partners from the inner and outer setting chosen to help address the barrier (*CFIR domain: inner and outer setting*), (3) the solutions elicited through interviews with these systems-level partners to address the barrier (*CFIR domain: process, construct: engage*), and (4) the adaptations made to the Asthma Link intervention based on this multi-level input (*CFIR domain: intervention adaptation*). These results demonstrate not only these CFIR domains discretely but also the influence that each domain has on other domains.

**Table 2:** CENTER-IT methodology to adapt intervention components: Asthma Link case study

***Individuals involved***	***Inner/outer setting***	***Process***	***Intervention adaptation***
*Step 1* (deliverer/recipient of interventions who identified barrier)	*Step 2* (systems-level partners selected by expert consensus group to address barrier)	*Step 3* (elicited solution to barrier)	*Step 4* (adaptation to intervention components based on expert consensus group and multi-level input)
**a. Barrier: Providers did not have time to determine if the patient was eligible, thus did not introduce program to eligible families**			
Medical providers	Practice managers	Practice managers offered to systematically identify and flag eligible patients for providers	Practice trainings now ask practice managers to systematically identify and flag eligible patients for providers
**b. Barrier: Family unable to pick up 2 inhalers from the pharmacy**			
Families School nurses Medical providers	Pharmacists	Pharmacists recommend the provider’s prescription should specify to dispense two inhalers.	Practice training teaches providers to specify on the prescription to “dispense 2 inhalers, one for home and one for school”
	Health insurers	Recommend establishing a Medicaid pharmacy policy to allow for 2 preventive inhalers to be dispensed at one time for Asthma Link patients	We are establishing an Asthma Link-specific policy with Medicaid pharmacy team to allow coverage of 2 inhalers for Asthma Link participants
**c. Barrier: Family unable to bring the 2nd inhaler to school**			
Families School nurses Medical providers	Pharmacists Practice managers	Recommends using free Mail-order delivery service to send medication from pharmacy to school	Practice trainings explain how to set up select families with mail-order delivery of medication from pharmacy to school
**d. Barrier: Delays with schools receiving faxed orders from practice staff**			
School nurses	Practice managers	Practice managers recommend changing the workflow so orders are faxed immediately	Practice staff faxes orders as soon as phone call with school nurse is complete
**e. Barrier: School nurses reported some school may not have a nurse to administer medications**			
School nurses	School administrators	Recommends identifying alternate professional at school capable of administering medications	Identify alternate officials in schools who can administer medications to children (e.g. health aid, counselor
**f. Barrier: School nurses reported some children did not consistently come to the nurse’s office**			
School nurses	School administrators	Recommends providing a list of Asthma Link patients to principals/teachers so they can facilitate bringing child to health office	School nurse will provide principal, teacher with list of Asthma Link patients
**g. Barrier: No support for daily asthma therapy during school breaks or holidays**			
School nurses Medical providers	Practice managers School administrators DPH Health insurers	Recommend developing a system to aid children during school breaks	Remote Asthma Link was created for when school is not in-session: daily text message to caregiver and remote weekly school health check-in
**h. Barrier: No established dissemination or sustainability protocol. At present school-supervised therapy is not widely adopted across the state or nation**			
School nurses Medical providers	Legislators DPH Health insurers	Recommend partnering current Asthma Link clinic/school leaders and new clinic/school leaders (targeting districts with high asthma rates) to facilitate knowledge and trust in Asthma Link Recommended presenting data on healthcare utilization outcomes and cost reduction to support reimbursement for Asthma Link	Partner clinical/school leaders currently participating in Asthma Link with new clinical/school leaders to share their Asthma Link experiences –(disseminate to districts with high asthma rates) Asthma Link team will present outcomes and cost savings data to payers, legislators, DPH

Key examples of the themes (recommended solutions) that emerged for specific constructs (barriers) are described below.

#### Identifying eligible patients

Medical providers (*individuals involved*) identified the barrier of not having enough time to identify whether a patient was eligible for Asthma Link during clinical encounters, and thus did not introduce the program to families. Based on this, we chose to interview practice managers (*inner setting*) who have a systems-view of the practice. Interviews with practice managers elicited the following solution (*process*): practice managers reported that they could systematically identify and flag potentially eligible patients using practice-level reports. We have incorporated this recommendation into the Asthma Link practice trainings, now asking practice managers to systematically identify and flag potentially eligible patients within their practice. (*intervention adaptation*).

#### Distribution of 2 inhalers

Caregivers of patients, medical providers, and school nurses (*individuals involved*) identified challenges with the patient being able to receive two preventive inhalers at one time from the pharmacy: one for home and one for school use for the intervention. Based on this, we chose to interview pharmacists who dispense the medication (*inner setting*) and Medicaid officials who often denied the coverage of two inhalers (*outer setting*). Interviews with pharmacists elicited the following solution (*process*): pharmacists recommended that providers write “please dispense 2 inhalers, one for home and one for school- Asthma Link patient” on the prescription. We have incorporated this recommendation into the Asthma Link practice training (*intervention adaptation*). Interviews with Medicaid officials elicited the following information and solutions (*process*): the current Medicaid pharmacy policy does not automatically allow two preventive inhalers to be dispensed at one time. Therefore, in the interview, Medicaid officials recommended creating a specific Medicaid pharmacy policy to ensure prescriptions labeled with “Asthma Link patient” are permitted to fill two inhalers at the pharmacy. This solution has been incorporated into the intervention protocol and we have worked with Mass Health (state Medicaid in Massachusetts) to create this policy such that 2 inhalers can now be dispensed at one time for patients labeled “Asthma Link patient” on the prescription (*intervention adaptation*).

#### Bringing inhaler medication to school

School nurses and medical providers (*individuals involved*) identified the challenge of families not bringing the inhaler medicine to the school. Based on this, we chose to interview pharmacists (*inner setting*). They recommended that families set up mail-delivery for this medicine to be sent directly to the school from the pharmacy, as this is a free service that most pharmacies provide (*process*). We have incorporated this into the Asthma Link training such that providers and clinic staff are taught to educate their patients about mail-delivery pharmacy services available (*intervention adaptation*).

#### Sending medication orders from physician’s offices to schools

School nurses (*individuals involved*) identified challenges with receiving medication orders from physicians’ offices in a timely fashion. Based on this, we chose to interview practice directors (*inner setting*) who recommended that practice staff send these orders immediately after completing the phone call with the school nurse (*process*). This new workflow has been incorporated into the practice training and Asthma Link intervention protocol (*intervention adaptation*).

#### Absence of a school nurse to supervise medication administration

School nurses (*individuals involved*) reported that some schools may not always have a school nurse present to supervise medication administration. Based on this, we chose to interview school principals (*inner setting*), who stated that schools always have a staff member capable of supervising medication administration. They recommended identifying such individuals at schools where full-time school nurses were not present (*process*). The school training for Asthma Link now includes a step to identify alternate professionals capable of supervising medication administration in these situations (*intervention adaptation*).

#### Ensuring children in Asthma Link go to the school health office daily

School nurses (*individuals involved*) identified challenges ensuring that all children enrolled in Asthma Link go to the school health office daily to receive their medicine. Based on this, we interviewed school principals (*inner setting*) who recommended making the list of Asthma Link patients available to the teachers and principals so both parties could assist with this process (*process*). We adapted the Asthma Link intervention such that the school nurse now provides a list of Asthma Link patients to teachers and the school principal (*intervention adaptation*).

#### Gaps in school-supervised therapy during school breaks

School nurses and medical providers (*individuals involved*) were concerned that children would experience gaps in school-supervised therapy during the summer and school holidays. Based on this, we interviewed practice directors, school leaders (*inner setting*), Department of Public Health (DPH), and insurance officials (*outer setting*) who all recommended that a system be created to support children’s medication adherence during school breaks (summer, holidays or during remote school conditions) (*process*). In response, we developed Remote Asthma Link as an adaptation to traditional Asthma Link (*intervention adaptation*). This is an automated text message system that sends caregivers of children in the program a daily reminder for their child’s preventive inhaler, shares the text responses with school nurses and then parents and children receive a remote asthma medication check-in with a school health official on a weekly basis when school is not in session. Separate studies of this Remote Asthma Link intervention are being conducted.

#### Asthma Link dissemination and sustainability

Medical providers, school nurses, and patients/caregivers (*individuals involved*) were concerned about sustainability and dissemination of Asthma Link to new clinical practices and school districts. In response, we interviewed practice directors, school leaders (*inner setting*), legislators, Department of Public Health, and insurance officials (*outer setting*) who recommended partnering current Asthma Link leaders in clinics/schools with new clinical/school leaders who have yet to participate in Asthma Link to promote buy-in and trust in the program (*process*). They advised focusing on dissemination to school districts with high asthma rates. To facilitate sustainability of Asthma Link, health insurers (*outer setting*) recommended the presentation of data on healthcare utilization (improvements in emergency room visits and hospital admissions) and cost savings to payers and policymakers (*process*). The Asthma Link protocol now includes a dissemination and sustainability plan including the partnership of existing and new clinical/school leaders as well as a sustainability plan which includes the presentation of healthcare utilization and cost savings data to payers and policymakers (*intervention adaptation*).

## Discussion

Guided by the CFIR, and through an iterative process of multi-level partner engagement, our study team developed a novel methodology to center multi-level partner voices (including those of providers, patients, and caregivers) into systems-level adaptations for the Asthma Link intervention. The CENTER-IT methodology demonstrates an empirical use of the CFIR and helps clarify the relationship between CFIR domains. Moreover, it shows the dynamic interplay between individuals involved in EBPs and the organization and context in which they work.

Through the Asthma Link case example, we showcase the CENTER-IT methodology, wherein we first elicit barriers identified by the individuals involved on the ground level of interventions and present them to systems-level partners who are well positioned to develop solutions, informing tailored intervention adaptations. The CENTER-IT approach informs the development of systems-level adaptations to interventions that are meaningful to partners at all levels (patient, caregiver, provider, and system).

The CFIR provides overarching typology to promote implementation theory; however, there is a dearth of simple reproducible examples and models for its meaningful, in-depth and pragmatic use [28]. Many implementation science frameworks, including the CFIR, focus on assessing the outer context and systems-level factors that are critical for implementation; however, the provider and patient/caregiver perspectives are not central to this process. Moreover, these frameworks have not provided clarifications on how domains or levels can interact and be synergistic. The results of the present study demonstrate not only the CFIR domains discretely but also the influence that each domain has on other domains. We employed a simple and novel methodology for the pragmatic use of the CFIR, which centers multi-level partners in systems-level changes and shows the dynamic interactions between the domains of this framework. While we applied our approach to the CFIR, it could be applied to any implementation theory or framework which recommends multi-level partner engagement to facilitate the adoption of evidence-based strategies.

The methodological approach presented in this study provides guidance to researchers on how to practically address provider and patient/caregiver-identified implementation barriers as well how to select and engage systems-level partners to address these barriers. The inclusion of these multi-level perspectives in intervention adaptation contrasts with the traditional researcher-led intervention design that often falls short of producing effective implementation in practice. This approach has the potential to identify solutions to key implementation barriers. For example, we identified the Asthma Link patient-identified barrier of “obtaining two inhalers” in step 1, then selected to interview pharmacists and Medicaid officials in step 2, elicited their recommended solutions to address this barrier in step 3, and established a MassHealth pharmacy policy to facilitate insurance coverage for two inhalers and trained providers to write “dispense two inhalers” on prescriptions in step 4. Since developing these systems-level adaptations, 100% of patients enrolled in Asthma Link for the 2020–2021 school year were able to obtain 2 preventive inhalers at one time. Noting the growing body of literature on intervention adaptations in implementation science, we must consider the feasibility, acceptability as well as intended and unintended consequences of adaptations to leverage best practice from research, so as to not compromise factors such as intervention fidelity and appropriateness [29, 30]. Future work will closely examine the feasibility, acceptability, and impact of these protocol adaptations through retrospective application of the Model for Adaptation Design and Impact [29] and detailed assessment of implementation outcomes in a hybrid effectiveness-implementation trial of the adapted Asthma Link protocol.

There are several strengths to this study. First, we developed a simple model of participatory implementation science and co-creation of an adapted intervention, strategies that are key to effective knowledge translation [31]. This model not only builds on an existing implementation science framework but can also be applied to other implementation theories or frameworks to promote the uptake of EBPs. Second, we demonstrated a novel and empiric use of a widely accepted implementation framework, starting upstream in the research process at the intervention design and adaptation phase, addressing an identified gap in implementation science [28]. Third, we meaningfully engaged partners at multiple levels (patient, provider, organizational, payer and policy) with the goal of advancing health equity by supporting improved uptake of EBPs by patients and families most in need. This heeds the recommendations of the National Institutes of Health, Patient Centered Outcomes Research Institute, and multiple implementation science frameworks [5]. The limitations include the small sample size; however, we stopped interviews at thematic saturation according to the tenets of qualitative research and our sample size is outweighed by the unique and impactful input from these stakeholders. Herein, we focused on one intervention, and it would be useful to see the CENTER-IT approach applied to other evidence-based practices.

## Conclusions

As we strive to advance implementation science, through more meaningful use of implementation frameworks and amplification of participant voices, this study demonstrates the use of the CENTER-IT methodology to engage with multi-level partners to facilitate these processes. The CENTER-IT methodology anchors the engagement of multi-level partners to the voices of the individuals who deliver and receive evidence-based interventions. The goal of this methodology is to understand multi-level implementation determinants and inform adaptations to EBPs that reflect participant perspectives and improve implementation. Future work should evaluate the extent to which multi-level partner engagement, using the CENTER-IT methodology, produces intervention adaptations that improve implementation of evidenced-based practices.

## Data Availability

The data that support the findings of this study are available on request from the corresponding author [MT]. The data are not publicly available due to their containing information that could compromise the privacy of research participants.

## Abbreviations

CFIR: Consolidated Framework for Implementation Research
EBPs: Evidenced-based practices
